# Tumor-stroma ratio as a new prognosticator for pseudomyxoma peritonei: a comprehensive clinicopathological and immunohistochemical study

**DOI:** 10.1186/s13000-021-01177-1

**Published:** 2021-12-13

**Authors:** Ru Ma, Yu-Lin Lin, Xin-Bao Li, Feng-Cai Yan, Hong-Bin Xu, Zheng Peng, Yan Li

**Affiliations:** 1 Department of Peritoneal Cancer Surgery, Beijing Shijitan Hospital, Capital Medical University, No. 10 Tieyi Road, Yangfangdian Street, Haidian District, 100038 Beijing, China; 2 Department of Pathology, Beijing Shijitan Hospital, Capital Medical University, 100038 Beijing, China; 3 Department of Myxoma, Aero Space Central Hospital, Peking University, 100049 Beijing, China; 4 Department of General Surgery, Chinese PLA General Hospital, 100853 Beijing, China

**Keywords:** Pseudomyxoma peritonei, Tumor-stroma ratio, Histopathology, Immunohistochemistry, Prognosis

## Abstract

**Background:**

As a rare clinical tumor syndrome with an indolent clinical course and lack of pathognomonic symptoms, pseudomyxoma peritonei (PMP) is usually diagnosed at an advanced stage. In-depth pathological analysis is essential to assess tumor biological behaviors, assist treatment decision, and predict the clinical prognosis of PMP. The tumor-stroma ratio (TSR) is a promising prognostic parameter based on the tumor and stroma. This study explored the relationship between TSR and the pathological characteristics and prognosis of PMP.

**Methods:**

PMP patients with complete data who underwent cytoreductive surgery plus hyperthermic intraperitoneal chemotherapy were enrolled. The TSR of postoperative pathological images was quantitatively analyzed by Image-Pro Plus. Then the relationship between TSR and the clinicopathological characteristics, immunohistochemical characteristics and prognosis of PMP was analyzed.

**Results:**

Among the 50 PMP patients included, there were 27 males (54.0%) and 23 females (46.0%), with a median age of 55 (range: 31–76) years. 25 (50.0%) patients were diagnosed with low-grade PMP (LG-PMP), and 25 (50.0%) were diagnosed with high-grade PMP (HG-PMP). There were 4 (8.0%) patients with vascular tumor emboli, 3 (6.0%) patients with nerve invasion, and 5 (10.0%) patients with lymph node metastasis. The immunohistochemical results showed that the Ki67 label index was < 25% in 18 cases (36.0%), 25 - 50% in 18 cases (36.0%) and > 50% in 14 cases (28.0%). The range of TSR was 2 - 24% (median: 8%). The cutoff value of TSR was 10% based on the receiver operating characteristic (ROC) curve and X-Tile analysis. There were 31 (62.0%) cases with TSR < 10% and 19 (38.0%) cases with TSR ≥ 10%. The TSR was closely related to histopathological type (*P* < 0.001) and Ki67 label index (*P* < 0.001). Univariate analysis showed that preoperative carcinoembryonic antigen (CEA), preoperative carbohydrate antigen 19–9, pathological type, vascular tumor emboli and TSR influenced the prognosis of PMP patients (*P* < 0.05). Multivariate analysis showed that preoperative CEA, vascular tumor emboli and the TSR were independent prognostic factors.

**Conclusions:**

The TSR could be a new independent prognosticator for PMP.

## Background

Pseudomyxoma peritonei (PMP) is a malignant clinical syndrome characterized by the accumulation and redistribution of copious mucus produced by mucinous tumor cells in the peritoneal cavity, with typical clinical manifestations including mucinous ascites, peritoneal implantation, omental cake, and ovarian involvement [1]. Currently, cytoreductive surgery (CRS) plus hyperthermic intraperitoneal chemotherapy (HIPEC) is the standard treatment, which can significantly prolong survival [2]. As a rare clinical tumor syndrome with an indolent clinical course and lack of pathognomonic symptoms, PMP is difficult to diagnose in the early stage, leading to missed diagnosis and misdiagnosis. In addition, the Peritoneal Surface Oncology Group International (PSOGI) consensus on the pathological types of PMP in 2016 ended the controversy and set standards on pathological classification and diagnostic terms of PMP [3, 4].

The histopathological classification and grading of PMP are of vital importance for disease assessment. Based mainly on the number of tumor cells and morphology of tumor nests, atypia, mitotic figures, and the form of surrounding invasion, PMP is divided into four different prognostic groups: acellular mucin, low-grade PMP (LG-PMP), high-grade PMP (HG-PMP) and HG-PMP with signet ring cells [4].

Over the past decade, increasing attention has been given to the interaction between tumor cells and the microenvironment. The tumor microenvironment, also known as the tumor-associated matrix, is composed of immune cells, fibroblasts, pericytes, and endothelial cells in the extracellular matrix. Tumor invasion is a multifactorial process that is significantly affected by the tumor microenvironment, and the synergistic interaction between tumor cells and stromal components is the main driving force of tumor progression and metastasis [5]. The tumor-stroma ratio (TSR) reflects the area of tumor and stromal cells and is determined by histopathological sections stained with hematoxylin and eosin (HE). Currently, TSR has been proven to impact the prognosis of esophageal squamous carcinoma, lung cancer, breast cancer, gastric cancer, and other malignant solid tumors [6, 7]. This study analyzed the relationship between TSR and the histopathological and immunohistochemical characteristics of PMP and investigated the impact of TSR on PMP prognosis.

## Materials and methods

### Patient selection

We selected PMP patients who received CRS+HIPEC at our center from June 2015 to May 2020 and had complete clinical data. The data included clinicopathological characteristics, immunohistochemical characteristics and follow-up data. The selection criteria were as follows: (1) all the surgical specimens and HE stained slides were re-read by a senior pathologist majoring in PMP pathology (Yan FC), according to the PSOGI histopathology diagnostic criteria of PMP [4], and LG-PMP and HG-PMP slides derived from the appendix were selected; (2) neoadjuvant therapy was not performed before CRS+HIPEC; (3) the follow-up time was longer than 3 months; and (4) the clinical data were complete. The study was approved by the Ethics Committee of Beijing Shijitan Hospital, and all patients signed informed consent forms.

### Study parameters

The clinicopathological characteristics were sex, age, previous surgical history, body mass index (BMI), Karnofsky (KPS) score, preoperative serum tumor markers, histopathological type, peritoneal cancer index (PCI), completeness of cytoreduction (CC), vascular tumor emboli, nerve invasion, and lymph node metastasis.

The immunohistochemical characteristics were Ki67, p53, MUC1, MMR gene-related proteins (MLH1, MSH2, MSH6 and PMS2), CDX2, CK7 and CK20.

The survival was determined from the last CRS+HIPEC.

### CRS+HIPEC procedure

After general anesthesia, a midline incision was made from the xiphoid process to the pubic symphysis, and then the extent of peritoneal metastasis from the diaphragmatic peritoneum to the pelvic peritoneum was explored. The nature and amount of ascites and the location and size of the tumor were recorded in detail. On this basis, PCI was evaluated [8]. Subsequently, maximum CRS was performed, including removal of the primary tumor at the acceptable edge and any adjacent structures involved, lymph node resection, and peritonectomy (tumor involvement on the peritoneal surface), according to the peritonectomy procedures by Sugarbaker [9]. After CRS, CC was assessed based on the residual tumor size [8].

HIPEC was performed after CRS with open Colliseum technology. Each drug was dissolved in 3 L of heated saline at 43 ± 0.5 ℃ for 60 min at a flow rate of 400 mL/min. The main HIPEC protocol included 120 mg of cisplatin plus 120 mg of docetaxel.

### Quantitative analysis of TSR (Fig. 1)

**Fig. 1:**
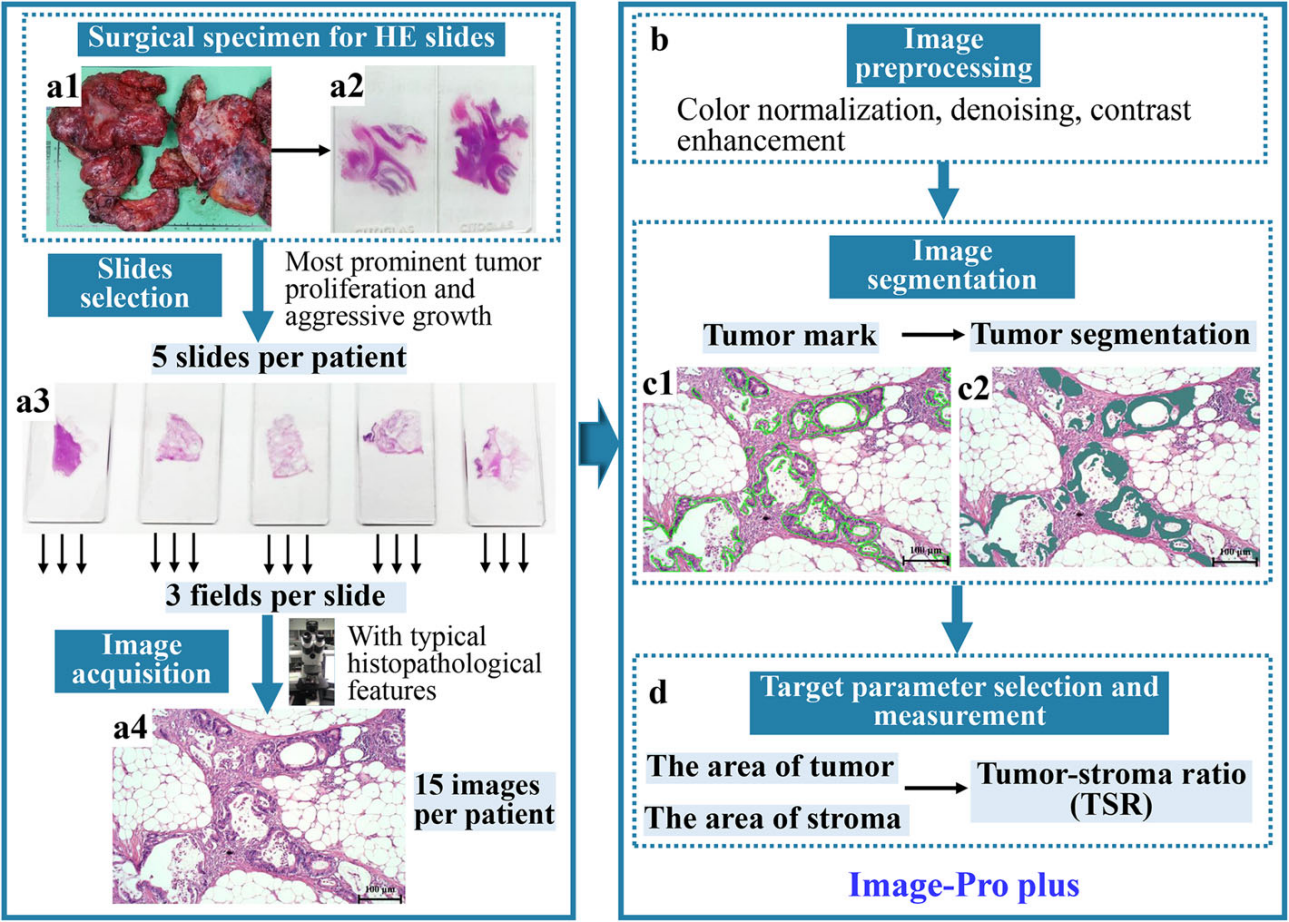
The workflow of histopathological quantitative analysis. **A1-2** Surgical specimen of PMP for HE staining slides; **A3** 5 HE stained slides with most prominent tumor proliferation and aggressive growth; **A4** Pathological image with typical histopathological features of PMP (HE staining, ×100); **C1-2** Tumor and stroma were segmented by Image-Pro Plus (HE staining, ×100). PMP: Pseudomyxoma peritonei; HE: Hematoxylin and eosin; TSR: Tumor-stroma ratio

The whole process of the quantitative analysis of TSR is as follows:

(1) Surgical specimen study for routine pathology: All the conventional surgical specimens of PMP patients undergoing CRS+HIPEC at our center were subjected to thorough histopathological study, with routine HE staining (Dako Hematoxylin, Dako Eosin and Dako Bluing Buffer, catalog number CS701; Dako CoverStainer, Agilent Technologies Inc., USA) (Fig. 1 A1, A2).

**Fig. 2:**
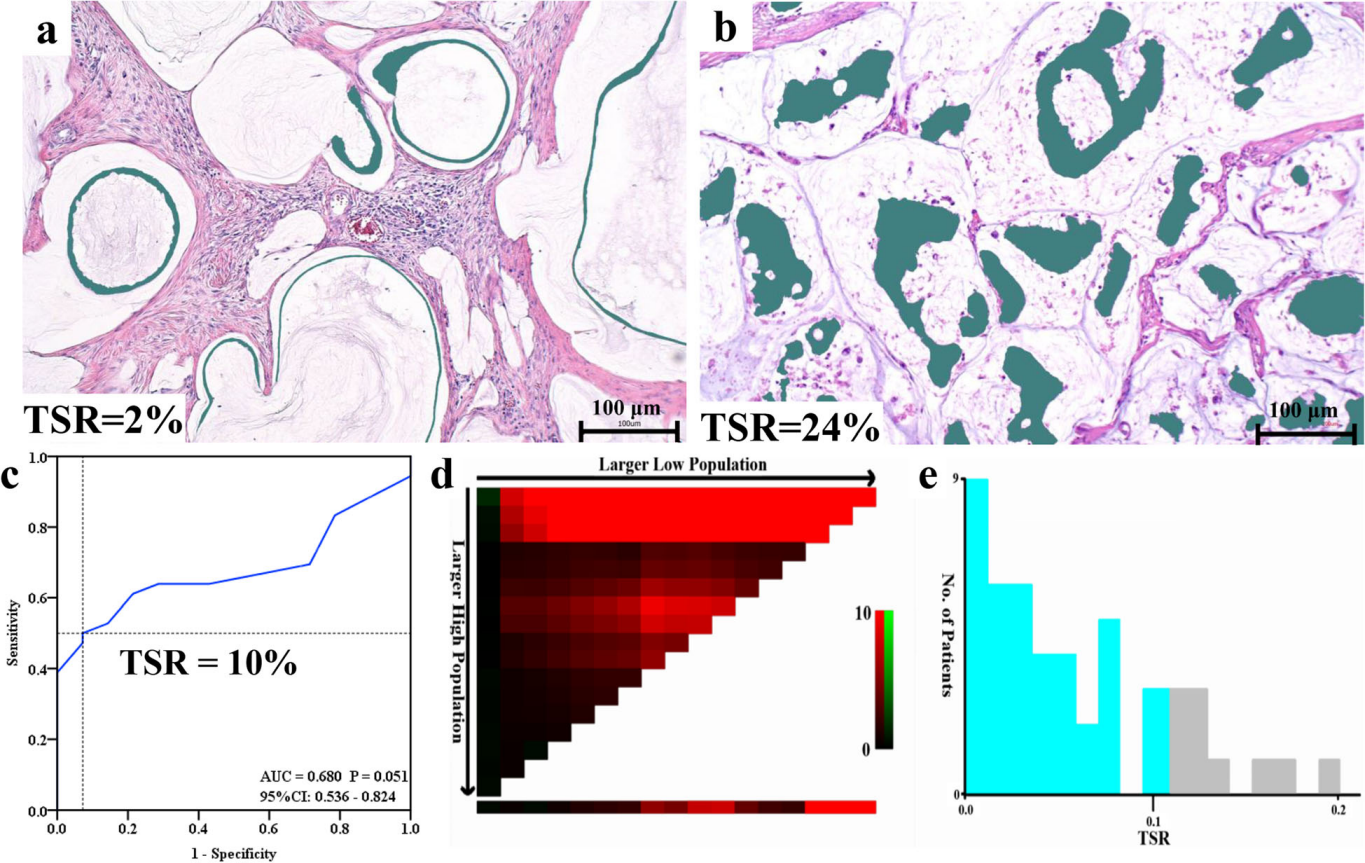
The description of TSR. **A** The pathological image of patient with minimum TSR of 2%; **B** The pathological image of patient with maximum TSR of 24% (A, B: HE staining, ×100); **C** ROC curve of TSR of 50 patients. The dotted line indicated that TSR of 10% was the best cutoff value with high sensitivity and specificity; **D, E**: The cutoff value of TSR obtained by X-Tile software. TSR: Tumor-stroma ratio; HE: Hematoxylin and eosin; ROC: Receiver operating characteristics

(2) Slides selection for the current study: For each specimen, 5 HE stained slides with most prominent tumor proliferation and aggressive growth, as interpreted by a senior clinical diagnostic pathologist majoring in PMP pathology (Yan FC), were selected from different parts of the specimen (Fig. 1 A3).

(3) Image acquisition: each of the 5 selected slides were again first overviewed and assessed under ×50 magnification, and 3 visual fields with typical histopathological features were randomly selected for image acquisition at ×100 magnification. Then 15 nonoverlapping JPG images with a resolution of 2048 × 2072 pixels were acquired from each specimen (Axio Scope.A1 biological microscope, Carl Zeiss AG, Germany; MS60 microscope camera, Mshot Technology Co., Ltd, China) (Fig. 1 A4).

(4) Image preprocessing: The above obtained images were first preprocessed to create unified images for subsequent quantitative evaluation, using Image-Pro Plus (IPP) 6.0 software (Media Cybernetics Inc., USA). The major techniques for image preprocessing included contrast enhancement, color normalization and denoising (Fig. 1B).

(5) Tumor-stroma segmentation and area measurement: The standardized images were segmented using IPP to separate the tumor area and the stroma area (Fig. 1C). The percentage of tumor area in each pathological image was measured, and the remaining area was the percentage of stroma. Thus, the ratio of tumor and stroma was calculated (Fig. 1D).

### Immunohistochemistry (IHC) analysis

IHC was performed on an intelliPATH FLX (BIOCARE MEDICAL LLC, USA) with a Polymer Immunohistochemical Detection System (Wuxi OriGene Technologies Inc., China). We analyzed the protein expression of Ki67 (clone UMAB107, OriGene, catalog number ZM-0166), p53 (clone DO7, OriGene, catalog number ZM-0408), MUC1 (clone MRQ-17, OriGene, catalog number ZM-0391),MLH1 (clone ES05, OriGene, catalog number G10090), MSH2 (clone 25D12, OriGene, catalog number G07855), MSH6 (clone EP49, OriGene, catalog number G07841), PMS2 (clone EP51, OriGene, catalog number G14519), CDX2 (clone EP25, OriGene, catalog number ZA-0520), CK7 (clone EP16, OriGene, catalog number ZM-0071), and CK20 (clone EP23, OriGene, catalog number ZA-0574) by IHC. All antibodies were ready-to-use. A positive control was set up according to the instructions, and phosphate buffer solution was used as a blank control.

The IHC images were interpreted as the following. The positive expression of Ki67, p53, mismatch repair (MMR) gene-related proteins, and CDX2 was in the cell nucleus, while mucin was in the cytoplasm, and CK7 and CK20 were in the cytoplasm and cytomembrane. The Ki67 labeling index was calculated as the number of positive cells/count cells × 100%. Wild-type p53 showed positive nuclear staining with different strengths and uneven distribution, while the mutant type showed strong positive nuclear staining with dense and uniform intensity. Any loss of MLH1, MSH2, MSH6, or PMS2 was a loss of MMR gene-related proteins. The presence of brown–yellow or tan granules in the tumor cells was defined as positive.

### Follow-up

The follow-up was once every 3 months within 2 years after CRS+HIPEC, once every 6 months for the third year, and once a year from the fourth year. The follow-up records mainly included general conditions, serum tumor markers, chest, abdomen, and pelvic enhanced CT + 3D reconstruction, and survival information. The last unified telephone or outpatient clinic follow-up was on Feb. 21, 2021, with a follow-up rate of 100%.

### Statistical analysis

All statistical analyses were performed using SPSS V24.0 statistical software (IBM SPSS Inc., USA). Age, BMI, KPS, and PCI score were expressed as medians (ranges), while the remaining features were expressed as rates or percentages. The relationship between TSR and pathological features was analyzed with the chi-square test. The Kaplan–Meier method and log-rank test were used for survival analysis. The cutoff values were obtained by receiver operating characteristic (ROC) curve and X-Tile software. *P* < 0.05 was considered as statistically significant.

## Results

### Demographic and clinical features of 50 patients in this study

A total of 50 patients were selected in the study, including 27 (54.0%) males and 23 (46.0%) females, with a median age of 55 (range: 31–76) years and a median KPS score of 90 (range: 60–100). 25 (50.0%) patients were diagnosed with LG-PMP, and 25 (50.0%) were diagnosed with HG-PMP. The median PCI score was 36 (range: 3–39); the CC score was 0–1 in 18 (36.0%) cases and 2–3 in 32 (64.0%) cases (Table 1).

**Table 1:** Major clinicopathological features of 50 PMP patients

Items	Value
Sex, n (%)	
Male	27 (54.0)
Female	23 (46.0)
Age (years), median (range)	55 (31 - 76)
BMI (kg/m^2^), median (range)	22.0 (15.9 - 27.8)
KPS score, median (range)	90 (60 - 100)
Operation History, n (%)	
No	7 (14.0)
Yes	43 (86.0)
History of intravenous chemotherapy, n (%)	
No	27 (54.0)
Yes	23 (46.0)
History of intraperitoneal chemotherapy, n (%)	
No	27 (54.0)
Yes	23 (46.0)
Preoperative CEA, n (%)	
Normal	9 (18.0)
Increased	41 (82.0)
Preoperative CA19-9, n (%)	
Normal	22 (44.0)
Increased	28 (56.0)
Preoperative CA125, n (%)	
Normal	22 (44.0)
Increased	28 (56.0)
Histopathological type, n (%)	
LG-PMP	25 (50.0)
HG-PMP	25 (50.0)
PCI score, median (range)	36 (3 - 39)
CC score	
0 - 1	18 (36.0)
2 - 3	32 (64.0)

PMP: Pseudomyxoma peritonei; BMI: Body mass index; KPS: Karnofsky performance status; LG-PMP: Low-grade PMP; HG-PMP: High-grade PMP; PCI: Peritoneal cancer index; CC: Completeness of cytoreduction; CEA: Carcinoembryonic antigen; CA19–9: Carbohydrate antigen 19–9; CA125: Carbohydrate antigen 125.

### Routine histopathological and immunohistochemical characteristics of 50 patients in this study

Among the 50 patients in this study, there were 25 cases each in the LG-PMP and HG-PMP groups. Other prominent histological features were vascular tumor emboli in 4 (8.0%) cases, nerve invasion in 3 (6.0%) cases, and lymph node metastasis in 5 (10.0%) cases.

IHC showed that the Ki67 label index was < 25% in 18 (36.0%) cases, 25-50% in 18 (36.0%) patients, and > 50% in 14 (28.0%) cases. The mutant rate of p53 was 62.0%. The expression rate of MUC1 was 74.3%. The loss rate of MMR gene-related protein expression was 6.7%. The expression rates of CDX2, CK7 and CK20 were 83.3%, 55.6% and 95.7%, respectively.

### TSR evaluation

After image segmentation and target-object calculation of 15 images for each patient specimen, the TSR was produced. The median TSR was 8% (range: 2 - 24%) (Fig. 2A, B). ROC curve and X-Tile analysis were used to obtain the cutoff value of TSR. Based on the ROC curve (Fig. 2C), at the cutoff value of 10%, the TSR reached 0.500 sensitivity and 0.929 specificity in predicting prognosis. In X-Tile analysis, we added the survival status, overall survival after last CRS+HIPEC, and TSR into the input data, and then the output results showed that the 10% was the optimal cutoff value of TSR (Fig. 2D, E). Therefore, the TSR was divided into < 10% and ≥ 10% for the subsequent analysis.

### The relationship between TSR and pathological features

The TSR was correlated with histopathological types and Ki67 (*P* < 0.001) but not with vascular tumor emboli, nerve invasion, lymph node metastasis, p53, MUC1, MMR protein expression, CDX2, CK7, and CK20 (*P* > 0.05) (Table 2).

**Table 2:** The relationship between TSR and pathological characteristics of PMP

Items	n (%)	TSR		*P*
		< 10%	≥ 10%	
Histopathological type				
LG-PMP	25 (50.0)	23	2	**< 0.001**
HG-PMP	25 (50.0)	8	17	
Vascular tumor emboli				
Yes	4 (8.0)	1	3	0.293
No	46 (92.0)	30	16	
Nerve invasion				
Yes	3 (6.0)	0	3	0.095
No	47 (94.0)	31	16	
Lymph node metastasis				
Yes	5 (10.0)	2	3	0.560
No	45 (90.0)	29	16	
Ki67 label index				
< 25%	18 (36.0)	17	1	**0.001**
25-50%	18 (36.0)	10	8	
≥ 50%	14 (28.0)	4	10	
p53				
Wild type	19 (38.0)	15	4	0.053
Mutant type	31 (62.0)	16	15	
MUC1				
+	26 (74.3)	16	10	1.000
-	9 (25.7)	5	4	
MMR protein expression				
Normal	28 (93.3)	15	13	0.464
Loss	2 (6.7)	0	2	
CDX2				
+	35 (83.3)	21	14	0.676
-	7 (16.7)	3	4	
CK7				
+	25 (55.6)	14	11	0.787
-	20 (44.4)	12	8	
CK20				
+	44 (95.7)	26	18	1.000
-	2(4.3)	1	1	

TSR: Tumor-stroma ratio; PMP: Pseudomyxoma peritonei; LG-PMP: Low-grade PMP; HG-PMP: High-grade PMP; MMR: Mismatch repair

### Survival analysis

#### Survival curve analysis

**Fig. 3:**
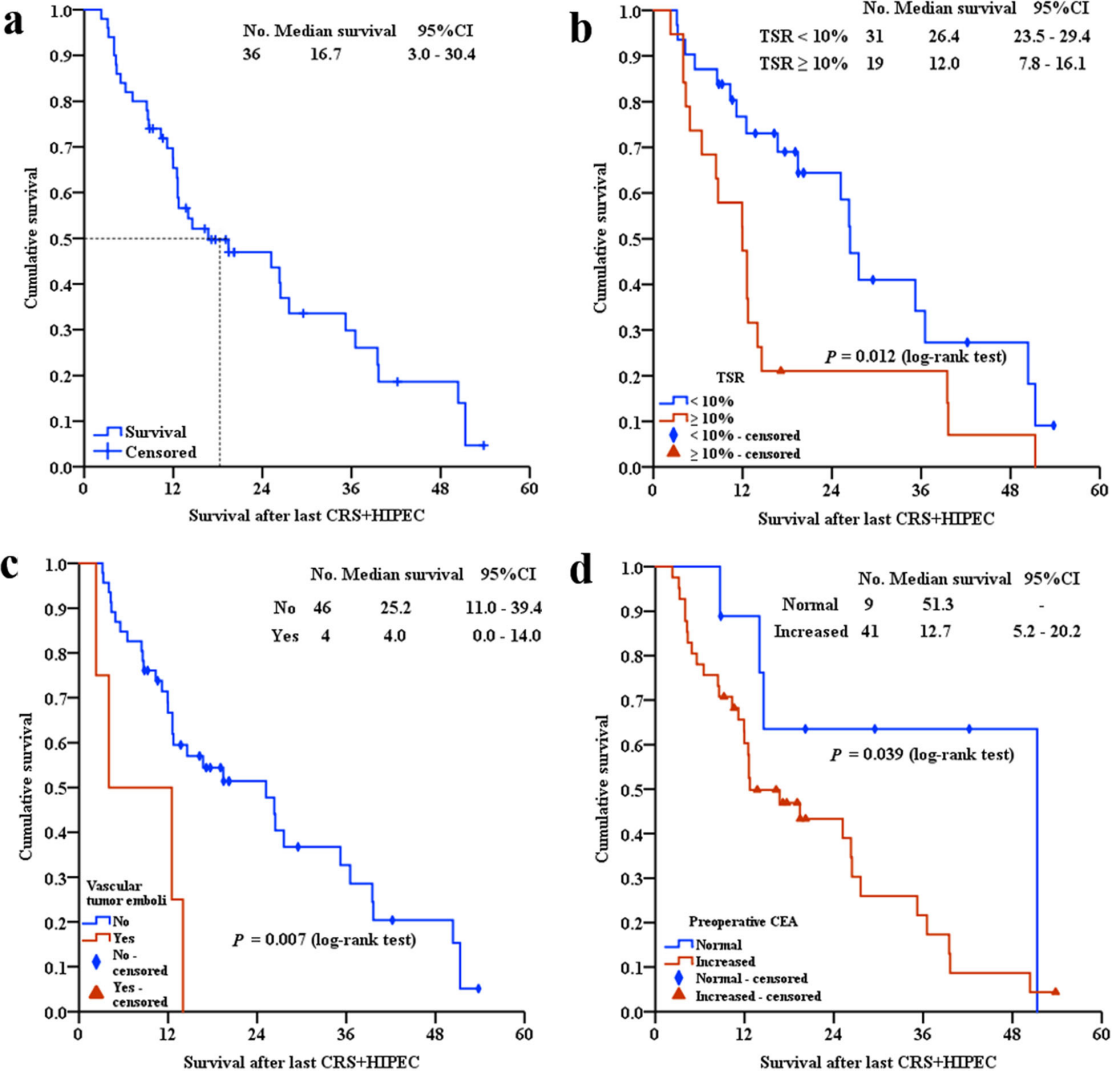
Survival curve and univariate analysis of 50 PMP patients. **A** Survival curve; **B** TSR; **C** Vascular tumor emboli; **D** Preoperative CEA. PMP: Pseudomyxoma peritonei; TSR: Tumor-stroma ratio

At the median follow-up time of 42.0 months (95% CI: 9.38–75.02 months), 36 (72.0%) patients died, 14 (28.0%) patients survived, and the median survival after the last CRS+HIPEC was 16.7 months (95% CI: 2.95–30.47 months). The 1-, 2- and 3-year survival rates were 65.4%, 47.0% and 29.8%, respectively (Fig. 3A).

#### Univariate analysis

Univariate analysis demonstrated the 5 factors with a statistically significant impact on survival after the last CRS+HIPEC: preoperative CEA (*P* = 0.039), preoperative CA19–9 (*P* = 0.032), pathological type (*P* = 0.022), vascular tumor emboli (*P* = 0.007), and TSR (*P* = 0.012).

#### Multivariate analysis

Factors with *P* < 0.05 in univariate survival analysis were included in the Cox regression model for multivariate analysis. In addition, PCI and CC, shown as independent prognostic factors in previous literature, were also included in the multivariate analysis, even though they were not statistically significant in the univariate analysis. Preoperative CEA, vascular tumor emboli and TSR were independent prognostic factors (Fig. 3B–D). The mortality risk for patients with elevated preoperative CEA was 4.091 times that of patients with normal preoperative CEA (*P* = 0.008, 95% CI: 1.441–11.616). The mortality risk for patients with vascular tumor emboli was 5.377 times that of patients without vascular tumor emboli (*P* = 0.004, 95% CI: 1.706–16.944). The mortality risk for patients with TSR ≥ 10% was 2.550 times that of patients with TSR < 10% (*P* = 0.007, 95% CI: 1.286–5.058) (Table 3).

**Table 3:** Multivariate survival analysis of 50 PMP patients

Variable	Wald	HR	95%CI	*P*
Preoperative CEA (Increased vs. Normal)	7.003	4.091	1.441 - 11.616	**0.008**
Preoperative CA19–9 (Increased vs. Normal)	-	-	-	0.112
PCI (≥25 vs. < 25)	-	-	-	0.925
CC (2-3 vs. 0-1)	-	-	-	0.521
Vascular tumor emboli (Yes vs. No)	8.250	5.377	1.706 - 16.944	**0.004**
Pathological types (HG-PMP vs. LG-PMP)	-	-	-	0.285
TSR (≥ 10% vs. < 10%)	7.179	2.550	1.286 - 5.058	**0.007**

PMP: Pseudomyxoma peritonei; LG-PMP: Low-grade PMP; HG-PMP: High-grade PMP; CEA: Carcinoembryonic antigen; PCI: Peritoneal cancer index; CC: Completeness of cytoreduction; TSR: Tumor-stroma ratio

## Discussion

In this study, TSR was obtained by quantitative analysis of HE histopathological images of PMP. We retrospectively analyzed the relationship between TSR and histopathological characteristics, immunohistochemical characteristics, and prognosis in 50 patients with complete clinicopathological information. Our results showed that TSR was closely related to histopathological types and Ki67 (*P* < 0.05). Multivariate survival analysis showed that preoperative CEA, vascular tumor emboli and TSR were independent prognostic factors.

As a rare clinical malignant tumor syndrome, the incidence of PMP is approximately 2–4 per million, and early diagnosis is difficult. Surgical resection is the main treatment. CRS+HIPEC has been recommended as the standard treatment of PMP internationally, which significantly improves the prognosis of patients [10]. However, approximately 1/3 of PMP patients treated with CRS+HIPEC will relapse even if complete CRS is achieved [11]. Therefore, accurate disease assessment is particularly important for treatment decisions and response evaluations. Currently, the internationally recognized pathologic classification of PMP is mainly based on the subjective qualitative evaluation of PMP pathological images by pathologists. The results are easily affected by the experience level of pathologists, the complexity of images and the visual search process, which leads to inaccurate pathological diagnosis. This showed that pathological type is an independent prognostic factor in PMP patients [12]. However, Mhhamed et al. [13] found that although most LG-PMP patients had a relatively better prognosis, some LG-PMP patients showed highly aggressive disease progression, with a 5-year survival rate much lower than those of patients with worse tumor differentiation. In addition, Baratti et al. [14] used PSOGI pathological classification to analyze the prognosis of 265 patients with PMP, and the results showed that acellular mucinous and HG-PMP-S were identified as subgroups with good prognosis and poor prognosis, respectively, but pathological classification was not an independent prognostic factor of PMP patients. These studies indicated that current pathological classification is not enough to accurately predict the prognosis of patients, and more in-depth and objective indicators need to be explored to improve the precision and clarity of pathological diagnosis. Few studies have been reported on the quantitative analysis of pathological images of PMP. Nevertheless, due to the rarity of PMP and the late start of research, pathological image-related research is still at the semiquantitative level, with stratified analysis based on morphological features only, so the calculation method is simple [15, 16].

Tumor invasion is a multifactor-driven process in which the synergistic interaction between tumor cells and stromal components is the driving force for tumor progression and metastasis. The tumor microenvironment is involved in tumorigenesis, progression, invasion, and metastasis by inducing stem cell-like characteristics and epithelial-mesenchymal transformation of tumor cells [17]. Therefore, the ratio of tumor and stroma can more accurately evaluate the biological behavior of tumors to a certain extent. TSR is the ratio of the tumor and stromal area, which can be obtained from pathological sections of routine HE staining of postoperative specimens without additional cost, so it is simple to operate and easy to apply. In recent years, the TSR has been proven to have prognostic value in many tumors. Vangangelt et al. [18] evaluated HE sections of 1794 patients with breast cancer and found that the TSR was not affected by clinically relevant factors such as age, tumor size and histology, and patients with low TSR had a relatively poor prognosis, which could be used as a potential prognostic indicator. Wu et al. [19] carried out a meta-analysis of 4238 cases of solid tumors, including in cervical cancer, nonsmall cell lung cancer, esophageal cancer, ovarian cancer, hepatocellular carcinoma, colorectal cancer, and nasopharyngeal carcinoma patients. Low TSR was significantly associated with advanced clinical stage, depth of invasion, and lymph node metastasis. Patients with high TSR were related to good clinical prognosis, and the TSR may be an independent prognostic factor for solid tumors [20].

Our study investigated the correlations between TSR and histopathological and immunohistochemical indicators of PMP. The TSR level of the LG-PMP group was significantly lower than that of the HG-PMP group. The TSR was positively correlated with Ki67, and patients with high Ki67 levels had a higher TSR. Previous studies have shown that the pathological type of PMP is an independent prognostic factor. A high-grade pathological type indicates the biological behavior of malignant and aggressive tumors, which is important for the selection of treatment regimens and prognosis evaluation of patients. Ki67 is a biological indicator reflecting the state of cell proliferation, and its expression changes with the cell cycle. The higher the value of Ki67, the more active the cell proliferation is, which has been proven to be a prognostic marker of tumors [21].

In recent years, the relationship between the pathological characteristics of PMP and survival prognosis has been gradually studied. Multiple studies have shown that histopathological type is an independent prognostic factor in PMP patients. Zhou et al. [12] conducted a meta-analysis of 766 PMP patients who underwent debulking and showed that LG-PMP had better survival than HG-PMP. Yan et al. [22] performed a clinicopathological and immunohistochemical analysis of 155 PMP patients undergoing CRS+HIPEC. Multivariate analysis showed that pathological type was an independent prognostic factor. Choudry et al. [23] retrospectively analyzed the tumor cell density of 310 PMP patients undergoing CRS+HIPEC and showed that the higher the cell density was, the shorter the progression-free survival.

In this study, 3 categories of indicators were included in this study: (1) sex, age, KPS score, preoperative serum tumor markers, PCI, CC and other systemic indicators; (2) pathological type, vascular tumor emboli, lymph node metastasis, Ki67, p53 and other traditional histopathological and immunohistochemical indicators; and (3) TSR. The results showed that preoperative CEA, vascular tumor emboli and TSR were independent prognostic factors. CEA is one of the most widely used serum tumor markers in clinical practice, which can assist in judging the degree of tumor invasion, and has important clinical value in disease detection and efficacy evaluation of gastrointestinal cancer and other malignant tumors. Carmignani et al. [24] analyzed the preoperative serum tumor markers of 532 patients with PMP and showed that elevated serum CEA at the time of recurrence indicated a poor prognosis, which could provide information related to disease progression. With the increase in CEA and CA19–9 in PMP patients after the second operation, the prognosis worsened. Vascular tumor emboli are the result of a series of pathological changes in the lymphatic and hematologic systems during tumor invasion and metastasis. They are closely associated with poor prognosis of various malignant tumors, including gastric cancer, colorectal cancer, and lung cancer [25]. Previous studies have shown that patients with a high cell density had a high risk of relapse [23], while our study shows that the TSR is an independent prognostic factor. The survival of patients with a high TSR is significantly shorter than that of patients with a low TSR; that is, the tumor area proportion is increased, and the prognosis of patients is worse, which is similar to previous studies. However, our study took stromal components into account, and analyzed the effects of both the tumor and stroma on the tumor biological behavior of PMP. A large amount of mucus aggregation in the stroma leading to intestinal obstruction is the main cause of death in PMP patients, and the study of the stroma is also of great significance for the prognosis of PMP patients [26].

Our study showed that patients with a low TSR had a better prognosis, which is contrary to other solid tumors. Several factors may account for the differences. First, previous studies on cellularity of PMP by Horvath et al. [15] and Choudry et al. [23] indicated that patients with scant and moderate cellularity were more likely to have recurrence and disease progression than those with acellular mucin, indicating that the larger the tumor cell density was, the stronger the invasion and metastasis of PMP [15]. In other words, the larger the tumor area, the more malignant the tumor biological behavior of PMP. Second, as a special clinical malignancy syndrome, PMP is characterized by copious mucus secretion, and a large amount of mucus accumulates in the stroma and envelops the tumor, thus significantly increasing the stromal area of PMP [27]. In addition, in a study integrating the “high-risk appendiceal cancer” (HRAC) signature and cancer-associated fibroblasts (CAFs) to predict the prognosis of PMP, it was found that good CAFs and HRAC showed good disease-free survival, demonstrating the importance of tumor stroma in PMP stratification [28]. However, a large amount of mucin secreted and occupied parts of the PMP stroma, which was a significant characteristic different from other solid tumors. Finally, due to the lack of quantitative pathological studies on PMP and the relatively small sample size in this study, the results may be limited to some extent, which is the weakness of this study. Future studies with large sample sizes are warranted to verify the findings from this study.

## Conclusions

This study found that the TSR was closely related to histopathological types and Ki67, two features of tumor aggression and proliferation, indicating that the TSR could help evaluate the biological behavior of PMP. Moreover, the TSR has a significant impact on PMP prognosis, suggesting that it could be a new prognostic indicator for PMP.

## Data Availability

The datasets used and/or analysed during the current study are available from the corresponding author on reasonable request.

## Abbreviations

PMP: Pseudomyxoma peritonei
TSR: Tumor-stroma ratio
LG-PMP: Low-grade PMP
HG-PMP: High-grade PMP
ROC: Receiver operating characteristic
CEA: Carcinoembryonic antigen
CA19–9: Carbohydrate antigen 19-9
CRS: Cytoreductive surgery
HIPEC: Hyperthermic intraperitoneal chemotherapy
PSOGI: Peritoneal Surface Oncology Group International
HE: Hematoxylin and eosin
IPP: Image-Pro Plus
IHC: Immunohistochemistry
MMR: Mismatch repair
BMI: Body mass index
KPS: Karnofsky
PCI: Peritoneal cancer index
CC: Completeness of cytoreduction
CA125: Carbohydrate antigen 125
